# Predicted Residual Error Sum of Squares of Mixed Models: An Application for Genomic Prediction

**DOI:** 10.1534/g3.116.038059

**Published:** 2017-01-19

**Authors:** Shizhong Xu

**Affiliations:** Department of Botany and Plant Sciences, University of California, Riverside, California 92521

**Keywords:** best linear unbiased prediction, cross-validation, generalized cross-validation, genomic selection, hybrid breeding, mixed model, Gen Pred, Shared data resource

## Abstract

Genomic prediction is a statistical method to predict phenotypes of polygenic traits using high-throughput genomic data. Most diseases and behaviors in humans and animals are polygenic traits. The majority of agronomic traits in crops are also polygenic. Accurate prediction of these traits can help medical professionals diagnose acute diseases and breeders to increase food products, and therefore significantly contribute to human health and global food security. The best linear unbiased prediction (BLUP) is an important tool to analyze high-throughput genomic data for prediction. However, to judge the efficacy of the BLUP model with a particular set of predictors for a given trait, one has to provide an unbiased mechanism to evaluate the predictability. Cross-validation (CV) is an essential tool to achieve this goal, where a sample is partitioned into *K* parts of roughly equal size, one part is predicted using parameters estimated from the remaining *K* – 1 parts, and eventually every part is predicted using a sample excluding that part. Such a CV is called the K-fold CV. Unfortunately, CV presents a substantial increase in computational burden. We developed an alternative method, the HAT method, to replace CV. The new method corrects the estimated residual errors from the whole sample analysis using the leverage values of a hat matrix of the random effects to achieve the predicted residual errors. Properties of the HAT method were investigated using seven agronomic and 1000 metabolomic traits of an inbred rice population. Results showed that the HAT method is a very good approximation of the CV method. The method was also applied to 10 traits in 1495 hybrid rice with 1.6 million SNPs, and to human height of 6161 subjects with roughly 0.5 million SNPs of the Framingham heart study data. Predictabilities of the HAT and CV methods were all similar. The HAT method allows us to easily evaluate the predictabilities of genomic prediction for large numbers of traits in very large populations.

Many diseases, anatomic structures, physiological characteristics, and behaviors in humans are polygenic traits. Most agronomic traits in agriculture, *e.g.*, yield, are also polygenic. These complex traits require whole-genome study to understand the genetic mechanisms and to genetically improve the quality and quantity of agricultural products (de los Campos *et al.* 2009, 2013a,b). Genomic prediction (selection) is a statistical method of whole-genome study (Meuwissen *et al.* 2001). It can lead to earlier detection of acute polygenic cancers (Vazquez *et al.* 2012). Genomic prediction is also an effective tool to select superior cultivars in plant breeding (Heffner *et al.* 2009). Genomic hybrid prediction will provide an opportunity to evaluate all potential hybrids and allow breeders to select superior hybrids that will have little chance to be discovered based on traditional hybrid breeding schemes (Xu *et al.* 2014). Genomic selection has been very successful in the dairy cattle industry (Goddard and Hayes 2007) and will soon become a routine procedure for breeding of a vast number of agricultural species.

Among the commonly used methods for genomic prediction, BLUP (Henderson 1975) is one of a few suitable methods for handling high-throughput genomic data with millions of genetic variants (VanRaden 2008). Reproducing kernel Hilbert spaces (RKHS) regression (Gianola *et al.* 2006) is another method with such an ability, but RKHS has not been as well recognized as the BLUP method. Although variable selection approaches such as Bayes B (Meuwissen *et al.* 2001) and LASSO (Tibshirani 1996) are optimal for traits with a few detectable loci of large effects plus many undetectable modifying loci under low and intermediate marker density, BLUP is the most robust method and one of the most commonly used genomic selection methods (de los Campos *et al.* 2013b). More importantly, the computational speed does not depend on marker density because it takes a marker-inferred kinship matrix (covariance structure) as the input data, albeit computing kinship matrix taking additional time. To evaluate the predictability of the BLUP model, one has to resort to some other tools, such as validation or CV, where individuals predicted do not contribute to estimated parameters that are used to predict these individuals. If individuals predicted are not excluded from the training sample, serious bias will occur in prediction.

The predictability of a model is often represented by the squared correlation coefficient between the observed and predicted phenotypic values (Xu *et al.* 2014). This squared correlation is approximately equal to $R^{2}=1-\text{PRESS}/SS$, where PRESS is the predicted residual error sum of squares and SS is the total sum of squares of the phenotypic values. Allen (1971, 1974) proposed to use PRESS as a criterion to evaluate a regression model, in contrast to using the estimated residual error sum of squares (ERESS) as the criterion. To calculate PRESS, Allen (1971, 1974) used an approach that is now called the leave-one-out cross-validation (LOOCV) or ordinary CV (Craven and Wahba 1979), in which an individual is predicted using parameters estimated from the sample that excludes this individual. When the sample size (*n*) is large, LOOCV presents a high computational cost because one will virtually have to analyze the data *n* times. The K-fold CV (Picard and Cook 1984) is an extension of LOOCV in which the sample is partitioned into *K* parts of roughly equal size. Individuals in a part are predicted simultaneously using all individuals in the remaining *K* − 1 parts. Eventually, all parts are predicted once and used to estimate parameters *K* − 1 times. When *K* is small, there are many different ways of partitioning the sample, leading to variation in the calculated predictability. This variation can be very large for small sample sizes. Therefore, people often repeat the K-fold CV a few times and use their average values to reduce the error due to random partitioning. If possible, LOOCV (also called the n-fold CV, a special case of K-fold CV when *K* = *n* and *n* is the sample size) is recommended because it eliminates all problems associated with this random partitioning variation. However, such a CV is not realistic for large samples under the mixed model methodology. Although a simple split CV (50% training and 50% test) should suffice with very large samples, still 50% of the sample is wasted. The LOOCV method may slightly overpredict the model compared with the K-fold CV when K is substantially smaller than *n* (Hastie *et al.* 2008).

Cook (1977, 1979) developed an explicit method to calculate PRESS by correcting the deflated residual error of an observation using the leverage value of the observation without repeated analyses of the partitioned samples. This method applies to least square regression under the fixed model framework, where the predicted *y* is a linear function of the observed *y* as shown below,

$$\hat{y}=X\hat{\beta }=X{\left(X^{T}X\right)}^{-1}X^{T}y=Hy$$ (1)

where

$$H=X{\left(X^{T}X\right)}^{-1}X^{T}$$ (2)

is called the hat matrix. The predicted residual error for observation *j* is $e_{j}={\hat{e}}_{j}/\left(1-h_{jj}\right)$ where ${\hat{e}}_{j}=y_{j}-X_{j}\hat{\beta }$ is the so-called estimated residual error and $h_{jj}$ is the leverage value of the *j*th observation (the *j*th diagonal element of the hat matrix). It is the contribution of the prediction for an individual from itself and may be called the conflict of interest factor. The predicted residual error is the estimated residual error after correction for the deflation. The sum of squares of the predicted residual errors over all individuals is the PRESS, which is a well-known statistic in multiple regression analyses. To find an explicit expression of PRESS for a mixed model, we need to identify a random effect version of the hat matrix and use the leverage value of the *j*th observation to correct for the deflated residual error.

The HAT method is a fast algorithm for the ordinary CV for a linear model (Allen 1971, 1974) because the regression analysis is only done once on the whole sample and then the estimated residual errors are modified afterward. Extension of the HAT method to mixed model has been made by Golab *et al.* (1979) in finding the optimal ridge factor in ridge regression (Hoerl and Kennard 1970a,b). It is well known that ridge regression can be formulated as a mixed model problem with the variance ratio replaced by a given ridge factor. Golab *et al.* (1979) proposed a generalized cross-validation (GCV) method to find the optimal ridge factor so that the generalized residual error variance is minimized. These authors showed that the GCV-calculated residual error sum of squares is a rotation-invariant version of Allen’s PRESS. The residual error variance obtained from the GCV method is equivalent to calculating the residual error variance by dividing the ERESS by an “effective” degree of freedom. Properties of the GCV method have been extensively studied by Li (1987). Jansen *et al.* (1997) applied GCV to wavelet thresholding. When performing genomic prediction, we prefer to see the actual predicted residual errors (errors in prediction of future individuals) obtained from the ordinary CV because the residual errors obtained via GCV may not be intuitive to most of us. The important gain from the GCV method of ridge regression analysis to genomic selection is the HAT matrix of the random model when the genomic variance is given.

There is rich literature on smoothing spline analysis that also helped us to develop the fast HAT method for evaluation of mixed model predictability (Wahba 1975, 1980, 1990, 1998; Wahba and Wold 1975a,b; Craven and Wahba 1979; Wahba *et al.* 1995, 2000; Wahba and Luo 1997; Wang 1998a,b; Hastie *et al.* 2008). In smoothing spline curve fitting, a response variable is fitted to a predictor with an arbitrary functional relationship. The common approach is to fit the curve using B-spline or another type of nonparametric approach. Several spline bases (more than necessary) are constructed from the original predictor. These bases are considered as new predictors, which are then used to fit the response variable with linear relationship. The regression coefficients are then estimated using a penalized shrinkage method such as the ridge regression. The ridge parameter in smoothing splines is then called the smoothing parameter (λ), which is often found so that the GCV residual error variance is minimized (Craven and Wahba 1979; Wahba 1980). Given the smoothing parameter, the predicted responses of all individuals are linear functions of all observed responses. Hastie *et al.* (2008) collectively called these linear functions the smoother matrix and denoted it by $S_{\lambda }.$ This smoother matrix is the random effect version of the HAT matrix,

$$H^{R}=X{\left(X^{T}X+\lambda Q\right)}^{-1}X^{T}$$ (3)

where *Q* is a known diagonal matrix. A HAT matrix under the random model was also given by de los Campos *et al.* (2013b) in the form of $\hat{y}={\left(G+\lambda I\right)}^{-1}y=Hy,$ although it was not derived for calculating PRESS. The HAT matrix of the fixed model introduced in Equation 2 is then denoted by $H^{F}.$ The difference between the two HAT matrices is clear in form. Hastie *et al.* (2008) stated that both $H^{R}$ and $H^{F}$ are symmetric and positive semidefinite, $H^{F}$ is idempotent ($H^{F}H^{F}=H^{F}$) but $H^{R}$ is not, and $H^{F}$ has a rank of *m* (number of predictors) while $H^{R}$ has a rank of *n* (number of observations). So, the HAT matrix for a random model has been defined by the smoothing splines community. We may implement this HAT matrix in our BLUP prediction to evaluate the predictability of our models and avoid the lengthy CV analysis. The smoothing parameter (our variance ratio) should be given a reasonable value and the REML estimate from the whole sample is a natural choice. However, replacing the prechosen λ by a data-driven estimate makes the HAT matrix a complicated function of the data. The question is, what is the difference between the HAT method (when λ is estimated from the whole sample) and the actual CV (when λ is estimated anew within each fold)? This becomes the main objective of this study.

When revising this manuscript, a similar study was published in the same journal (*G3: Genes| Genomes | Genetics*) by Gianola and Schon (2016). They also recognized the approximation nature of the new method and stated that using the whole-sample-estimated λ in place of the prechosen λ will not affect the result too much, especially when the LOOCV is compared, because the training sample only differs from the whole sample by one observation. However, this is only a speculation (most likely true) and they did not explicitly investigate the difference. Since the new method represents a significant technical improvement in genomic selection, the community must be aware of the difference before widely adopting the new method to evaluate a genomic selection program. In this study, we explicitly answer this question by analyzing several agronomic traits and 1000 metabolomic traits from two rice populations. Further comparison was also made in genomic prediction of human height from the Framingham heart study data (Dawber *et al.* 1951, 1963).

## Methods

### Fixed model

The HAT method for calculating PRESS under the fixed model is given by Cook (1977, 1979) for the LOOCV scenario but not for the leave $n_{k}$ out CV (the K-fold CV). We extended Cook’s method to leave $n_{k}$ out for weighted least squares regression analysis. The predicted *y* is a linear function of the observed *y* as shown below,

$$\hat{y}=X\hat{\beta }=X{\left(X^{T}WX\right)}^{-1}X^{T}Wy=Hy$$ (4)

where

$$H=X{\left(X^{T}WX\right)}^{-1}X^{T}W$$ (5)

is the hat matrix. This *H* matrix is still idempotent. In a K-fold CV analysis, let $n_{k}$ be the number of observations in the *k*th fold for k=1,…,K and $\sum_{k=1}^{K}n_{k}=n.$ Define $X_{k}$ as an $n_{k}\times p$ matrix of independent variables for individuals in the *k*th fold, where p is the number of independent variables. The “leverage” value for the *k*th fold is defined as an $n_{k}\times n_{k}$ matrix,

$$H_{kk}=X_{k}{\left(X^{T}WX\right)}^{-1}X_{k}^{T}W_{k}$$ (6)

where $W_{k}$ is the $n_{k}\times n_{k}$ subset of matrix *W* corresponding to the *k*th fold. This matrix must appear in the end, not in the beginning, of the above equation. Let

$${\hat{e}}_{k}=y_{k}-X_{k}\hat{\beta }$$ (7)

be the estimated residual errors where $\hat{\beta }$ is estimated from the whole sample. The predicted residual errors for the $n_{k}$ individuals in the *k*th fold is

$$e_{k}={\left(I-H_{kk}\right)}^{-1}{\hat{e}}_{k}.$$ (8)

Therefore, the PRESS is defined as

$$\text{PRESS}=\sum_{k=1}^{K}e_{k}^{T}W_{k}e_{k}={\hat{e}}_{k}^{T}{\left(I-H_{kk}\right)}^{-1}W_{k}{\left(I-H_{kk}\right)}^{-1}{\hat{e}}_{k}$$ (9)

which is the weighted sum of squares of the predicted residual errors. Derivation of Equation 9 is given in Appendix A.

### Mixed model

The linear mixed model for genomic prediction is written as

y=Xβ+ξ+e (10)

where Xβ represents the fixed effects, ξ is a vector of random (polygenic) effects with an assumed $N\left(0,A\sigma_{\xi }^{2}\right)$ distribution, and $e∼N\left(0,I\sigma^{2}\right)$ is a vector of residual errors. The expectation and variance of *y* are E(y)=Xβ and $\mathrm{var}\left(y\right)=V=A\sigma_{\xi }^{2}+I\sigma^{2},$ respectively, where *A* is a marker-inferred kinship matrix (explained in detail below), $\sigma_{\xi }^{2}$ is the polygenic variance, and $\sigma^{2}$ is the residual error variance. The parameters are $\theta =\left\{\beta ,\sigma_{\xi }^{2},\sigma^{2}\right\}$ and the variances are estimated using the restricted maximum likelihood method (Patterson and Thompson 1971) by maximizing the following likelihood function,

$$L\left(\theta \right)=-\frac{1}{2}\ln\left|V\right|-\frac{1}{2}\ln\left|X^{\text{T}}V^{-1}X\right|-\frac{1}{2}{\left(y-X\beta \right)}^{\text{T}}V^{-1}\left(y-X\beta \right).$$ (11)

The estimated genomic heritability (de los Campos *et al.* 2015) from the markers is ${\hat{h}}^{2}={\hat{\sigma }}_{\xi }^{2}/\left({\hat{\sigma }}_{\xi }^{2}+{\hat{\sigma }}^{2}\right).$ The best linear unbiased estimates (BLUE) of the fixed effects are $\hat{\beta }={\left(X^{\text{T}}V^{-1}X\right)}^{-1}X^{\text{T}}V^{-1}y$ and the BLUP of the polygenic effects are $\hat{\xi }={\hat{\sigma }}_{\xi }^{2}AV^{-1}\left(y-X\hat{\beta }\right).$ The fitted phenotypic values are $\hat{y}=X\hat{\beta }+\hat{\xi },$ which is a conditional prediction (not a marginal prediction). Corresponding to the predicted polygenic effect $\hat{\xi }=\hat{y}-X\hat{\beta },$ we now define $\xi =y-X\hat{\beta }$ as the “observed” polygenic effect (it is indeed observed because $\hat{\beta }$ is used). The model goodness of fit (FIT) for the random effects is defined as the squared correlation between ξ and $\hat{\xi }.$

### Marker-inferred kinship matrix

The marker-inferred kinship matrix *A* is calculated from all markers of the genome using the following equation,

$$A=\frac{1}{a}\sum_{k=1}^{m}Z_{k}Z_{k}^{\text{T}}$$ (12)

where *m* is the total number of markers, $a=n^{-1}\mathrm{tr}\left(\sum_{k=1}^{m}Z_{k}Z_{k}^{\text{T}}\right)$ is a normalization factor to make the diagonal elements of matrix *A* as close to unity as possible, and $Z_{k}$ is an n×1 vector of genotype indicator variables for all individuals at marker *k*. For individual *j*, the numerical code for a genotype is

$$Z_{jk}=\{\begin{array}{ccc} -1 & \text{for} & A_{1}A_{1}\\ 0 & \text{for} & A_{1}A_{2}\\ +1 & \text{for} & A_{2}A_{2} \end{array}$$ (13)

where $A_{1}A_{1},$ $A_{1}A_{2}$, and $A_{2}A_{2}$ are the three genotypes of the marker. People often standardize the $Z_{k}$ vectors before using them to calculate the kinship matrix (see VanRaden 2008).

### Cross validation

For a K-fold CV, we randomly partitioned the sample into *K* parts of roughly equal size. We then used K−1 parts to predict the remaining part. Let $y={\left[\begin{array}{cc} y_{k}^{\text{T}} & y_{-k}^{\text{T}} \end{array}\right]}^{\text{T}}$ be the vector of phenotypic values that are partitioned into $y_{k}^{\text{T}}$ and $y_{-k}^{\text{T}},$ where $y_{k}^{\text{T}}$ is a vector of phenotypic values of all observations in the *k*th part (test sample) and $y_{-k}^{\text{T}}$ is a vector of phenotypic values for all individuals excluding observations in the *k*th part (training sample). Corresponding to this partitioning of the sample, we have

$$\text{E}\left(y\right)=\left[\begin{array}{c} X_{k}\beta \\ X_{-k}\beta \end{array}\right]$$ (14)

and

$$\mathrm{var}\left(y\right)=V=\left[\begin{array}{cc} V_{kk} & V_{k\left(-k\right)}\\ V_{\left(-k\right)k} & V_{\left(-k\right)\left(-k\right)} \end{array}\right]=\left[\begin{array}{cc} A_{kk}\sigma_{\xi }^{2}+I\sigma^{2} & A_{k\left(-k\right)}\sigma_{\xi }^{2}\\ A_{\left(-k\right)k}\sigma_{\xi }^{2} & A_{\left(-k\right)\left(-k\right)}\sigma_{\xi }^{2}+I\sigma^{2} \end{array}\right].$$ (15)

The predicted phenotypic values in the test sample are

$$\text{E}\left(y_{k}|y_{-k}\right)={\hat{y}}_{k}=X_{k}{\hat{\beta }}_{-k}+\sigma_{\xi }^{2}A_{k\left(-k\right)}{\left(A_{\left(-k\right)\left(-k\right)}\sigma_{\xi }^{2}+I\sigma^{2}\right)}^{-1}\times (y_{-k}-X_{-k}{\hat{\beta }}_{-k}).$$ (16)

Let $\xi_{k}=y_{k}-X_{k}{\hat{\beta }}_{-k}$ be the “observed” polygenic effect (phenotypes of the test sample adjusted by the fixed effects or centered phenotypes) and

$${\hat{\xi }}_{k}^{CV}={\hat{\sigma }}_{\xi }^{2}A_{k\left(-k\right)}{\left(A_{\left(-k\right)\left(-k\right)}{\hat{\sigma }}_{\xi }^{2}+I{\hat{\sigma }}^{2}\right)}^{-1}\left(y_{-k}-X_{-k}{\hat{\beta }}_{-k}\right)$$ (17)

be the predicted polygenic effect for the test sample. After all parts of the sample are predicted, we calculate the PRESS using

$$\text{PRESS}=\sum_{k=1}^{K}{\left(\xi_{k}-{\hat{\xi }}_{k}^{CV}\right)}^{\text{T}}\left(\xi_{k}-{\hat{\xi }}_{k}^{CV}\right).$$ (18)

The predictability is defined as

$$R_{\text{CV}}^{2}=1-\text{PRESS}/\text{SS}$$ (19)

where

$$\text{SS}=\sum_{k=1}^{K}{\left(\xi_{k}-\bar{\xi }\right)}^{\text{T}}\left(\xi_{k}-\bar{\xi }\right)$$ (20)

is the total sum of squares of *y* adjusted by the fixed effects.

### The HAT method

With the HAT method, we first defined the adjusted or centered phenotypic vector by the fixed effects $\xi =y-X\hat{\beta }$ as the “observed” ξ and define $\hat{\xi }=\hat{y}-X\hat{\beta }$ as the predicted ξ, where $\hat{\beta }$ is estimated from the whole sample. We then used the whole sample to predict the polygenic effects

$$\hat{\xi }={\hat{\sigma }}_{\xi }^{2}AV^{-1}\left(y-X\hat{\beta }\right)={\hat{\sigma }}_{\xi }^{2}AV^{-1}\xi .$$ (21)

Comparing the second form of the above equation ($\hat{\xi }={\hat{\sigma }}_{\xi }^{2}AV^{-1}\xi$) with the fixed model HAT function, $\hat{y}=Hy,$ we realized that $\hat{\xi }=H^{\text{R}}\xi ,$ where $H^{\text{R}}={\hat{\sigma }}_{\xi }^{2}AV^{-1}$ is the HAT matrix of the random effects. Substituting $V^{-1}$ by ${\left(A\sigma_{\xi }^{2}+I\sigma^{2}\right)}^{-1}$ and after a few steps of algebraic derivation leads to

$$H^{\text{R}}={\hat{\sigma }}_{\xi }^{2}A{\left(A\sigma_{\xi }^{2}+I\sigma^{2}\right)}^{-1}={\left(I+\lambda A^{-1}\right)}^{-1}$$ (22)

where $\lambda =\sigma^{2}/\sigma_{\xi }^{2}$ is the variance ratio. With eigen-decomposition for the *A* matrix, we have $A=UDU^{\text{T}},$ $A^{-1}=UD^{-1}U^{\text{T}}$ and $UU^{T}=U^{T}U=I.$ Therefore,

$$H^{\text{R}}=U{\left(I+D^{-1}\lambda \right)}^{-1}U^{\text{T}}=U{\left(U^{T}U+\lambda D^{-1}\right)}^{-1}U^{\text{T}}.$$ (23)

This expression (the second form) is exactly the one defined by Hastie *et al.* (2008) for the smoothing spline analysis given in Equation 3, where their *X* is replaced by the eigenvector *U*, their *Q* is replaced by the inverse of eigenvalue matrix $D^{-1}$ (diagonal), and their smoothing parameter is our variance ratio. The HAT matrix is easy to compute because ${\left(I+\lambda D^{-1}\right)}^{-1}$ is diagonal. When some eigenvalues are zero, $D^{-1}$ does not exist (very often), we reformulate it by $D{\left(D+\lambda I\right)}^{-1}.$ Therefore,

$${\left(I+\lambda D^{-1}\right)}^{-1}=D{\left(D+\lambda I\right)}^{-1}=\mathrm{diag}\left\{\frac{\delta_{1}}{\delta_{1}+\lambda },\frac{\delta_{2}}{\delta_{2}+\lambda },\cdots ,\frac{\delta_{n}}{\delta_{n}+\lambda }\right\}$$ (24)

where $\delta_{j}$ is the *j*th eigenvalue of matrix *A*. Although the HAT method does not need to refit the model for each part predicted, it still needs to partition the sample into *K* parts if comparison with the traditional CV is of interest. Let ${\hat{e}}_{k}=\xi_{k}-{\hat{\xi }}_{k}$ be the estimated residual errors for all individuals in the *k*th part and $H_{kk}^{\text{R}}$ be the diagonal block of matrix $H^{\text{R}}$ corresponding to all individuals in the *k*th part. The predicted residual errors for the *k*th part are $e_{k}={\left(I-H_{kk}^{\text{R}}\right)}^{-1}{\hat{e}}_{k}.$ Proof of this predicted residual error is provided in Appendix B. The PRESS under this random model becomes

$$\text{PRESS}=\sum_{k=1}^{K}e_{k}^{\text{T}}e_{k}=\sum_{k=1}^{K}{\hat{e}}_{k}^{\text{T}}{\left(I-H_{kk}^{\text{R}}\right)}^{-2}{\hat{e}}_{k}.$$ (25)

The predictability is measured by

$$R_{\text{HAT}}^{2}=1-\text{PRESS}/\text{SS}$$ (26)

where $\mathrm{SS}=\sum_{k=1}^{K}{\left(\xi_{k}-\bar{\xi }\right)}^{T}\left(\xi_{k}-\bar{\xi }\right)$ is the total sum of squares for the centered *y* (adjusted by the fixed effects). The n-fold HAT approach is a special case where the *k*th part to be predicted contains only one individual, *i.e.*, $H_{kk}^{\text{R}}=h_{jj}^{\text{R}}$ for k=j. Therefore, the leave-one-out version of the PRESS is

$$\text{PRESS}=\sum_{j=1}^{n}e_{j}^{2}=\sum_{j=1}^{n}{\hat{e}}_{j}^{2}/{\left(1-h_{jj}^{\text{R}}\right)}^{2}.$$ (27)

This predictability is roughly equal to the squared correlation between the fixed-effect-adjusted phenotypes and the predicted polygenic effects. The ERESS is $\text{ERESS}=\sum_{j=1}^{n}{\hat{e}}_{j}^{2}$ and the usual R-square reported in regression analysis is $R^{2}=1-\text{ERESS}/\text{SS},$ which is a measurement of model FIT, not predictability.

### Generalized cross validation

GCV (Golab *et al.* 1979) is an alternative method to correct the deflated residual error variance. The GCV-calculated residual error sum of squares is called generalized residual error sum of squares (GRESS), which is defined by

$$\text{GRESS}=\frac{{\left(\xi -\hat{\xi }\right)}^{T}\left(\xi -\hat{\xi }\right)}{{\left[n^{-1}\mathrm{tr}\left(I-H^{R}\right)\right]}^{2}}$$ (28)

where $\hat{\xi }$ is the predicted polygenic effect from the whole sample. It is equivalent to dividing each estimated residual error by the average $\left(1-h_{jj}\right)$ across all observations. Therefore, an intuitive expression of the above equation is

$$\text{GRESS}=\sum_{j=1}^{n}{\hat{e}}_{j}^{2}/{\left(1-\bar{h}\right)}^{2}$$ (29)

where $\bar{h}=\sum_{j=1}^{n}h_{jj}/n$ is the average leverage value across all observations and ${\hat{e}}_{j}=\xi_{j}-{\hat{\xi }}_{j}.$ The predictability is defined as

$$R_{\text{GCV}}^{2}=1-\text{GRESS}/\text{SS}.$$ (30)

Golab *et al.* (1979) stated that GRESS is a rotation-invariant PRESS. It is not intuitive to interpret GRESS and therefore we prefer to report PRESS and thus $R_{\text{HAT}}^{2}.$

### Data availability

All data analyzed in this study have been previously published. Sources of these data are provided by the references cited in the text. The Framingham Heart Study Data were downloaded from NCBI dbGaP with an IRB number HS -11-159. The rice data along with the R codes are provided in Supplemental Files S1, S2, S3, S4, S5 and S6. Description of the supplemental files can be found in File S7.

## Results

### Properties of the HAT method

Properties of the HAT method will be demonstrated using an experimental rice population consisting of 210 recombinant inbred lines (Yu *et al.* 2011). These lines were derived from the cross of two rice varieties. A total of 270,820 SNPs were used to infer breakpoints of the genome for each line, resulting in a total of 1619 bins. A bin is a haplotype block within which there are no breakpoints across the entire population. In the original analysis of Yu *et al.* (2011), each bin was treated as a genetic marker. In this study, we used all the 1619 bins to infer a 210 × 210 kinship matrix. The matrix represents the genetic relationships of the lines and is used to model the covariance structure of the polygene. The population size is reasonably small and enabled us to compare the HAT method with CV in great detail.

Seven agronomic and 1000 metabolomic traits were included in the analysis. The agronomic traits are yield per plant (YD), tiller number per plant (TP), grain number per panicle (GN), 1000-grain weight (KGW), grain length (GL), grain width (GW), and heading day (HD). The first four traits (YD, TP, GN, and KGW) were field evaluated four times (two locations in 2 yr), and GL and GW were replicated twice (two different years), and HD was replicated three times (three different years). The phenotypic value of each trait for each line is the average of the replicates. The 1000 metabolites were measured from seeds (317) and leaves (683) with two biological replications (Gong *et al.* 2013). The phenotypic values of the metabolites are the average expression levels of the two replicates after log2 transformation.

**Figure 1:**
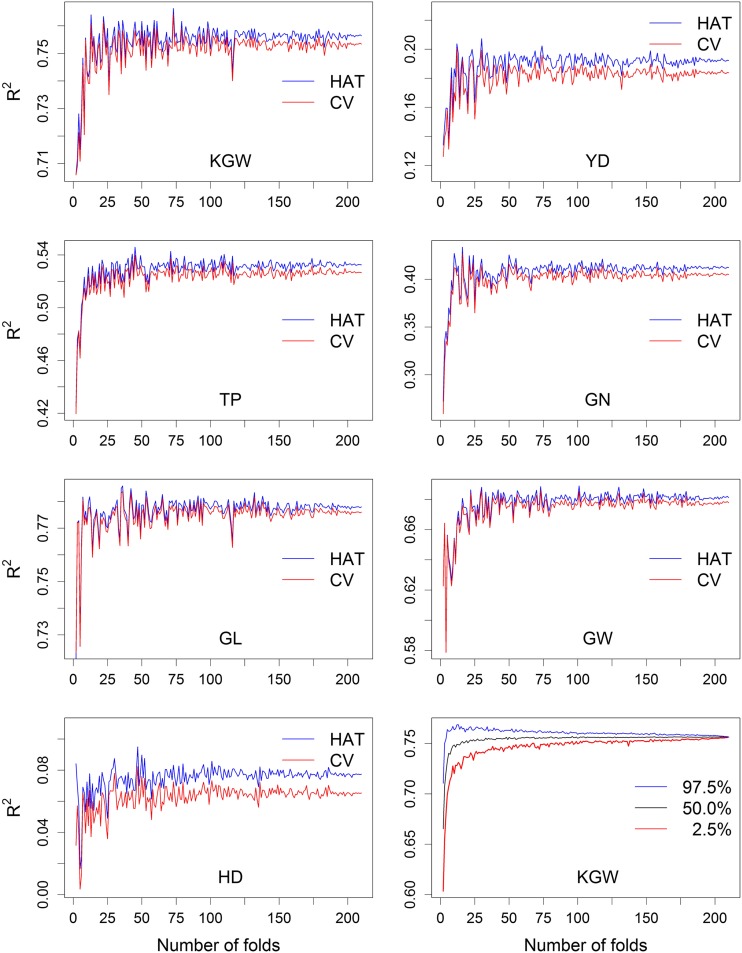
Comparison of predictabilities of the HAT and CV methods for seven agronomy traits in inbred rice. The first seven panels are the predictabilities of the HAT (blue) and CV (red) for the seven traits. The last panel shows the average predictabilities and the 95% confidence band of the HAT method for KGW from 100 random partitionings of the sample.

Predictability of the HAT method was compared with that of the CV method starting at twofold and ending at n-fold incremented by one, as shown in Figure 1 for the seven agronomic traits. The two methods produced very similar values of *R*-squares, with a slight upward bias for the HAT method due to the use of λ estimated from the whole sample. The biases are quite small for high predictability traits, *e.g.*, KGW and GL. They appear to be large for low predictability traits such as YD and HD. However, this is partly due to the small scale of the *y*-axis (a visual effect). For example, the predictabilities of HAT and CV for trait KGW are 0.7564 and 0.7534, respectively, and the corresponding predictabilities for trait HD are 0.0774 and 0.0653. Figure 1 also shows that when the numbers of folds are small, the predictabilities vary wildly and the variation progressively reaches zero at n-fold. The variation is caused by the ways that the folds are partitioned within the sample. Therefore, when a low number of folds are used in CV, it is necessary to repeat the CV a few times to reduce this variation. Although multiple CV will cause extra computational time, the HAT method can easily evaluate this variation.

Since computing the HAT method is sufficiently fast, we were able to perform random partitioning of the sample 100 times within a few minutes for all folds running from 2 to *n*. The last panel of Figure 1 shows the mean and 95% confidence band for the replicated HAT predictability for trait KGW. The average predictability reaches a plateau at ∼10-fold, but the 95% band is still very wide. This result did not support the claim that the LOOCV seriously biased the predictability compared with K-fold CV (Hastie *et al.* 2008).

**Figure 2:**
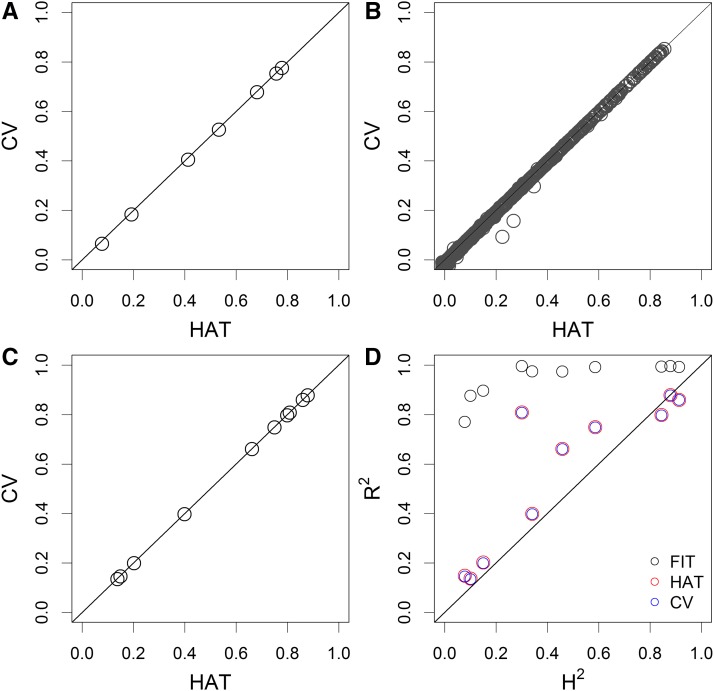
Predictability of the cross-validation (CV) method plotted against that of the HAT method under n-fold CV for different traits. (A) Seven traits of the inbred rice. (B) 1000 metabolomic traits of the inbred rice. (C) 10 traits in hybrid rice. (D) Plot of the *R*^2^ of the CV method, the HAT method, and the goodness of fit (FIT) against the estimated heritability (H^2^) obtained from replicated experiments for 10 agronomy traits of the hybrid rice.

Figure 2A shows the plot of predictability from n-fold CV against that from HAT for the seven agronomic traits of rice. The differences between the two methods are visually indistinguishable. We then compared the two methods for the 1000 metabolomic traits with n-fold CV. The CV method took a few days to complete the n-fold CV but the HAT method, again, took no more than a few minutes. The corresponding plots for the 1000 metabolomic traits are shown in Figure 2B. Except for three outliers, all points fall on the diagonal line. The three outliers show that the HAT prediction is overoptimistic.

### Genomic hybrid prediction in rice

We used a hybrid population of rice (Huang *et al.* 2015) to demonstrate the application of the HAT method to genomic hybrid breeding. The population consists of 1495 hybrid rice with 10 agronomic traits measured in two locations in China (Hangzhou and Sanya). The 10 traits are YD, panicle number (PN), GN, seed setting rate (SSR), KGW, HD, plant height (PH), panicle length (PL), GL, and GW. The phenotypic value of each hybrid is the average of the two locations. We used 1.6 million SNPs to infer the kinship matrix and then performed predictions using both the HAT and CV methods. Although the n-fold sample partitioning can be easily accomplished with the HAT method, it would be too costly to do it with the CV method. Therefore, we compared the two methods under the 10-fold CV. We replicated the experiment 20 times per 10-fold CV to reduce the variation caused by random partitioning of the sample. The average of the 20 replicates presents the predictability for each method.

The two replications of hybrid rice experiments allowed us to estimate trait heritability of the hybrid population using the traditional ANOVA method (Table 1). We partitioned the phenotypic variance into variance due to hybrids (genotypes) and variance due to residual error with systematic difference between the two locations excluded from the phenotype.

**Table 1:** Analysis of variance table to estimate heritability of agronomic traits from replicated experiments of hybrid rice

Source	Degree of Freedom	SS	MS	E(MS)*^a^*
Hybrids	1495 − 1 = 1594	SS_G_	MS_G_	$\sigma_{E}^{2}+2\sigma_{G}^{2}$
Locations	2 − 1 = 1	SS_R_	MS_R_	$\sigma_{E}^{2}+1495\varphi_{R}^{2}$
Residual errors	(1995 − 1) (2 − 1) = 1494	SS_E_	MS_E_	$\sigma_{E}^{2}$
Corrected total	2989	SS_T_	MS_T_	

SS, sum of squares; MS, means squares; E(MS), expected mean squares.

a These variance components are used to estimate the trait heritability $H^{2}=\sigma_{G}^{2}/\left(\sigma_{G}^{2}+\sigma_{E}^{2}\right).$

First, we compared the predictability of the CV method with the HAT method. Figure 2C shows the plot of the CV-generated predictability against the HAT-generated predictability. All the 10 points (one point per trait) fall on the diagonal line, indicating very good agreement between the two methods. We then compared the trait heritability (H2) from the two replicated environments with the predictability drawn from 10-fold CV, the predictability obtained from the HAT method (HAT), and the FIT. The plots are illustrated in Figure 2D. The *R*^2^ of HAT and CV are the same (the red circles overlap with the blue circles). Both HAT and CV fall around the diagonal line with some upward biases compared to H2. The FIT are severely biased upwards and are not good representatives of H2 at all.

**Figure 3:**
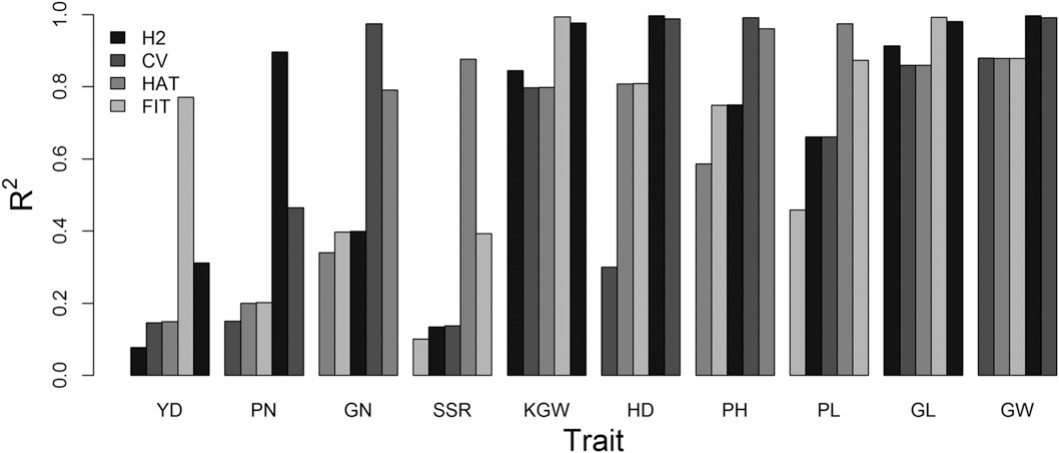
Comparison of *R*^2^ of the estimated heritability from replicated experiments (H2), the cross-validation (CV) method, the HAT method, and the model goodness of fit (FIT) for 10 traits of the hybrid rice.

Figure 3 shows a side-by-side comparison of H2 (trait heritability), *R*^2^ of HAT, CV, and FIT for all 10 traits, where FIT is equivalent to genomic heritability (de los Campos *et al.* 2015). Different traits have very different H2, ranging from 0.08 (YD) to 0.92 (GL). The difference between HAT and CV is virtually zero across all traits and both are higher than H2 for the majority of the traits. For the three highly heritable traits (KGW, GL, and GW), the H2 is higher than or equal to HAT and CV. Interestingly, HAT and CV are substantially higher than H2 for HD.

### Prediction of human height

We analyzed human height of 6161 subjects from the Framingham heart study (Dawber *et al.* 1951, 1963) with ∼0.5 million SNPs using the mixed model methodology incorporating the marker-inferred kinship matrix. The model included effects of generation (two levels) and gender (male and female) as fixed effects. The estimated polygenic and residual variances are ${\hat{\sigma }}_{\xi }^{2}=9.2375$ and ${\hat{\sigma }}^{2}=1.2617,$ respectively, yielding a $\hat{\lambda }=0.1365897$ and an estimated genomic heritability of ${\hat{h}}^{2}=0.8798.$ This genomic heritability is close to the reported gender average heritability of human height (0.75–0.88) (Silventoinen *et al.* 2003). The 10-fold CV and the HAT method gave predictabilities of 0.3063±0.0079 and 0.3151±0.0037, respectively. Note that the predictabilities are the averages of 20 replicated random partitions and thus there are small SEs associated with the average values. The predictability obtained from the leave-one-out HAT method is 0.3278, slightly higher than the 10-fold partitioning approach.

### GCV and optimization of λ

**Figure 4:**
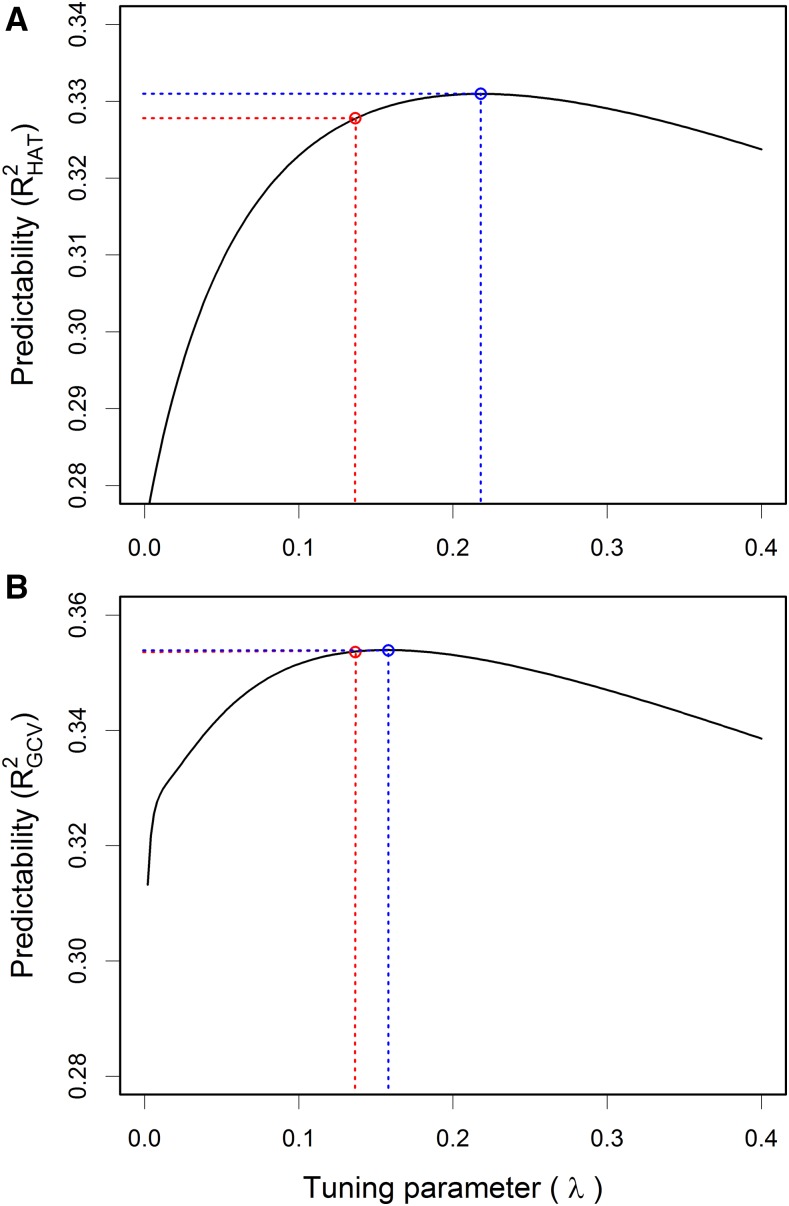
Tuning parameter ($\lambda =\sigma^{2}/\sigma_{\xi }^{2}$) that maximizes the genomic predictability ($R_{}^{2}$) of human height. (A) predictability profile of the HAT method, where the red point represents the predictability ($R_{\text{HAT}}^{2}=0.3278$) when the tuning parameter takes the REML estimate ($\hat{\lambda }=0.1366$) and the blue point represents the maximum achievable predictability ($R_{\text{HAT}}^{2}=0.3310$) when the tuning parameter is λ=0.2180. (B) predictability profile of the GCV method, where the red point represents the predictability ($R_{\text{GCV}}^{2}=0.3536$) when the tuning parameter takes the REML estimate ($\hat{\lambda }=0.1366$) and the blue point represents the maximum predictability ($R_{\text{GCV}}^{2}=0.3539$) when the tuning parameter is λ=0.1580.GCV, generalized cross-validation; REML, restricted maximum likelihood.

Before we perform the following analysis, it is worthwhile to refresh our mind that the HAT method will slightly overestimate the predictability because of the approximation nature. We first used the human height trait as an example to demonstrate the difference between the HAT method and the GCV method. The REML estimate of the variance ratio is $\hat{\lambda }=0.1366$ and the corresponding predictability from the n-fold HAT method is $R_{\text{HAT}}^{2}=0.3278.$ This REML estimate generates a GCV predictability of $R_{\text{GCV}}^{2}=0.3536,$ different from that of the HAT method. We now treated λ as a tuning parameter to maximize the predictability, as done by Mathew *et al.* (2015) in GCV for estimating breeding values. Using a grid search around the REML-estimated value ($\hat{\lambda }=0.1366$), we found that the maximum achievable predictability for the HAT method is $R_{\text{MAX}}^{2}=0.3310$ when λ=0.218, leading to a gain of 0.3310−0.3278=0.0032, which represents a (0.3310−0.3278)/0.3278≈1% gain in predictability. Although this gain is negligible, it demonstrates that the REML-estimated parameter does not give the maximum predictability. The good news is that $\hat{\lambda }$ is almost optimal, at least in this example. The corresponding maximum achievable predictability in GCV is $R_{\text{MAX}}^{2}=0.3539$ when λ=0.158, leading to a gain of 0.3539−0.3536=0.0003. Figure 4 shows the predictability profiles around $\hat{\lambda }=0.1366.$ By tuning the parameter, the gain in predictability of the HAT method (Figure 4A) is visible but the gain of the GCV method (Figure 4B) is not recognizable.

**Figure 5:**
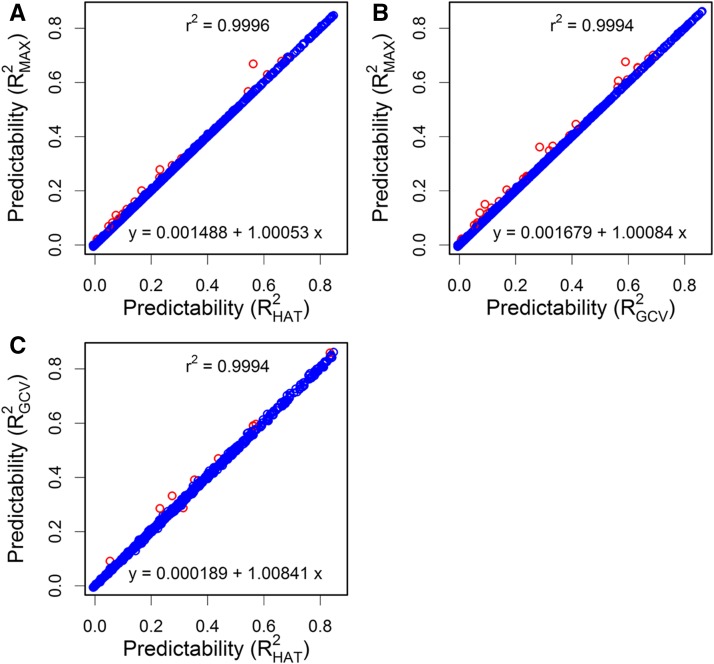
Comparison of predictability of REML-estimated λ with the maximum achievable predictability by tuning λ for 1000 metabolomic traits of rice. (A) Maximum achievable predictability of the HAT method by tuning λ plotted against the predictability when λ takes the REML estimate. (B) Maximum achievable predictability of the GCV method by tuning λ plotted against the predictability when λ takes the REML estimate. (C) Predictability of the GCV method plotted against the predictability of the HAT method when λ takes the REML estimate. The red points indicate traits with visible differences in predictability between the method shown on the *x*-axis and the method shown on the *y*-axis. The linear regression equation is given at the bottom of each panel and the fitted $r^{2}$ of the regression is given at the top of each panel. GCV, generalized cross-validation; REML, restricted maximum likelihood.

To further compare the predictabilities of the HAT and GCV methods with their maximum achievable predictabilities, we used the “Brent” method of the “opim()” function in R to search for the optimal tuning parameter (λ) for all 1000 metabolomic traits in the inbred rice population (210 lines). These optimal values of λ may be called the maximum predictability estimates (MPE). Figure 5 illustrates the comparisons of predictabilities across all 1000 traits, where more than a dozen traits show visible gains in predictability by tuning the parameter around the REML-estimated value for the HAT method (Figure 5A). Similar comparison is shown in Figure 5B for the GCV method where tuning the parameter achieves more than 20 visible gains in predictability. Figure 5C compares the predictabilities of GCV and HAT when the tuning parameter is fixed at the REML-estimated value. The two methods provided very similar predictabilities for all 1000 traits except a half dozen traits with visible differences. All three comparisons shown in Figure 5 have fitted *R*-squares at ∼0.9995 and the regression coefficients are not significantly different from one (*P* > 0.05) except C, where the regression coefficient is significantly > 1 (*P* < 0.05).

## Discussion

Very recently, Gianola and Schon (2016) published methods that are very similar to our HAT method to evaluate the predictability of a genomic selection model. They also recognized the approximation nature of the method when the smoothing parameter λ is replaced by the estimated value from the whole sample. Their justification of the use of this whole sample-estimated parameter, particularly in LOOCV, is that the estimated λ from the whole sample will not be much different from the ones obtained from the training samples that differ from the whole sample by just one observation. They actually investigated the variation of λ across all training samples and found that the variance is indeed small. Gianola and Schon (2016) investigated the properties of the new methods in many different situations using an inbred population of wheat (*n* = 599) to see how the predictability changes when the training and test sample size ratio changes. These exhaustive investigations would take months or years to complete if the ordinary CV were carried out. In addition to BLUP, these authors also extended the method to RKHS (Gianola *et al.* 2006) and the Bayesian alphabetic series (Gianola 2013) by modifying the importance sampling schemes.

One important issue that was not addressed in Gianola and Schon (2016) is how much difference in predictability calculated between the fast method and the classical CV method can be expected. This question is fundamental because the new method represents a significant technical improvement in genomic selection and will be adopted widely soon after the GS community recognizes it. In our study, we particularly focused on this question and investigated the difference using seven traits from an inbred population of rice, 1000 metabolomic traits from the same inbred population, 10 traits from a hybrid population of rice, and one trait (human height) from a large human population. We found that the HAT method always provides a slightly biased predictability over that of the CV method. However, the bias is never sufficiently severe to distort the conclusion on the predictability of a model. For example, in the human height prediction, the 10-fold CV produced a predictability of 0.3063 and the corresponding number from the 10-fold HAT method was 0.3151. However, the model FIT is 0.8789. The HAT method gave a number much closer to the CV predictability than the model FIT.

In addition to comparing the differences between the HAT method and the ordinary CV, we also compared the new HAT method with the GCV method (Golab *et al.* 1979) and found that the two produced very similar results. Craven and Wahba (1979) compared GCV with CV and concluded that the smoothing parameter that maximizes the CV was amazingly close to the parameter that maximizes GCV. The GCV method has been available for almost four decades, but the genomic selection community, except Mathew *et al.* (2015), has never paid attention to it. Our study showed that both GCV and HAT can be applied to genomic selection. However, the HAT method directly addresses prediction of future individuals and therefore it is more intuitive to interpret the result.

Hastie *et al.* (2008) claimed that LOOCV provides a biased prediction compared with CV with lower number of folds. We observed that when the number of folds is 10 or above, the predictability stabilizes (Figure 1, last panel). We did not observe a progressive increase of the predictability as the number of folds increases. Therefore, from our study, we recommend to perform LOOCV with the HAT method to avoid variation caused by random partitioning of the samples when the number of folds is small. When 10-fold or fivefold CV is carried out, the analysis will only be conducted 10 or 5 times, which may not be significant; therefore, the HAT method may lose its appeal. This statement may not be true considering the fact that the 10-fold CV must be run many times to reduce the variation caused by random partitioning of the samples. A multiple CV analysis for large samples is a significant burden to investigators. Therefore, the HAT method is a good alternative to CV to evaluate a genomic selection program.

We originally hoped to see a significant improvement in predictability by tuning the smoothing parameter around the REML-estimated parameter. It is disappointing that there were very few significant improvements from predictions of 1000 traits. The largest improvement occurred for the 422th metabolite with an improvement of (0.6683−0.5615)/0.5615=0.1902048≈19% (see the red point most deviating away from the diagonal line in Figure 5A). The good news is that, in most cases, the REML estimate is close to the MPE and, therefore, the parameter does not need to be tuned. On the other hand, since the computation is simple and fast, why not go ahead to tune the parameter and, if lucky, we may get an improved predictability, like the 422th metabolite in the inbred rice population.

In mixed model prediction, the random effects are often the targets for prediction. This is the case in genomic prediction because the genetic values are treated as random effects. However, if the investigators are interested in prediction using the fixed effects only under the mixed model, the estimated marginal residual error needs to be adjusted by the leverage values from the fixed model hat matrix $H^{F}=X{\left(X^{\text{T}}V^{-1}X\right)}^{-1}X^{\text{T}}V^{-1}$ (Schabenberger 2004). Let ${\hat{e}}_{k}=y_{k}-X_{k}\hat{\beta }$ be the estimated marginal residual errors for individuals in the *k*th fold, the predicted marginal residual errors are approximated by $e_{k}={\left(I-H_{kk}^{\text{F}}\right)}^{-1}{\hat{e}}_{k},$ where $H_{kk}^{\text{F}}$ is the diagonal block of $H^{\text{F}}$ corresponding to observations in the *k*th fold. The MIXED procedure in SAS calls this method the noniterative influence diagnostics while the iterative influence diagnostics is through actual CV (refit model and reestimate covariance parameters). The noniterative and iterative influence diagnostics can be interpreted as the HAT method and the CV method, respectively. PROC MIXED does not provide influence diagnostics for prediction of random effects. If there is an interest in both the fixed and random effects for prediction, the HAT matrix should include both the fixed model part and the random model part of the HAT matrix, $H^{\text{M}}=H^{\text{F}}+H^{\text{R}}\left(I-H^{\text{F}}\right).$ The estimated conditional residual errors are ${\hat{e}}_{k}=y_{k}-X_{k}\hat{\beta }-{\hat{\xi }}_{k}$ and the predicted conditional residual errors are obtained by $e_{k}={\left(I-H_{kk}^{\text{M}}\right)}^{-1}{\hat{e}}_{k},$ where $H_{kk}^{\text{M}}$ is the diagonal block of $H^{\text{M}}$ corresponding to observations in the *k*th fold.

When the mixed model includes multiple covariance structures, say *S* covariance structures, a similar $H^{R}$ matrix is used except that the $\sigma_{\xi }^{2}A$ and V matrices in $H^{R}$ are replaced by $G=\sum_{s=1}^{S}A_{s}\sigma_{s}^{2}$ and $V=\sum_{s=1}^{S}A_{s}\sigma_{s}^{2}+I\sigma^{2},$ respectively, where $A_{s}$ is the *s*th covariance structure and $\sigma_{s}^{2}$ is the corresponding variance. An example of the multiple variance component model is the model with nonadditive variances that include dominance and epistasis (Xu 2013). Gianola and Schon (2016) also extended the new method to handle multiple kernels.

The HAT method applies to fixed models (exact result) and linear mixed models (approximate result). Is it possible to extend the HAT method to LASSO and PLS (partial lest squares)? An approximate extension may be possible by fixing the shrinkage parameter, like the extension to BLUP, but there is no exact extension. To carry out that approximate extension, we need to find the HAT function of the predicted *y* on the observed *y*, *e.g.*, $\hat{y}=H^{\text{LASSO}}y$ and $\hat{y}=H^{\text{PLS}}y.$ In general, the HAT matrix is $H=\partial \hat{y}/\partial y$ (Schabenberger 2004), a Jacobian matrix holding each derivative of a predicted quantity with respect to an observed response.

## Supplementary Material

Supplemental material is available online at www.g3journal.org/lookup/suppl/doi:10.1534/g3.116.038059/-/DC1.

## Appendix A

### Predicted residual error sum of squares (PRESS)

In a K-fold CV analysis, let $n_{k}$ be the number of observations in the *k*th fold for k=1,…,K and $\sum_{k=1}^{K}n_{k}=n.$ Define $X_{k}$ as an $n_{k}\times p$ matrix of independent variables for the individuals in the *k*th fold. The “leverage” values for the *k*th fold is defined as an $n_{k}\times n_{k}$ matrix,

$$H_{kk}=X_{k}{\left(X^{T}WX\right)}^{-1}X_{k}^{T}W_{k}$$ (A1)

where $W_{k}$ is the $n_{k}\times n_{k}$ subset of matrix *W* corresponding to the *k*th fold. This matrix must appear in the end, not in the beginning, of the above equation. Let

$${\hat{e}}_{k}=y_{k}-X_{k}\hat{\beta }$$ (A2)

be the estimated residual errors where $\hat{\beta }$ is estimated from the whole sample. The predicted residual errors for the $n_{k}$ individuals in the *k*th fold is

$$e_{k}={\left(I-H_{kk}\right)}^{-1}{\hat{e}}_{k}$$ (A3)

Therefore, the PRESS is defined as

$$\text{PRESS}=\sum_{k=1}^{K}e_{k}^{T}W_{k}e_{k}={\hat{e}}_{k}^{T}{\left(I-H_{kk}\right)}^{-1}W_{k}{\left(I-H_{kk}\right)}^{-1}{\hat{e}}_{k}$$ (A4)

which is the weighted sum of squares of the predicted residual errors.

### Derivation of PRESS

The linear model for *p* independent variables (including the intercept) and *n* observations is

y=Xβ+e. (A5)

The weighted least squares estimates of all regression coefficients are obtained using

$$\hat{\beta }={\left(X^{T}WX\right)}^{-1}X^{T}Wy.$$ (A6)

The estimated residual error variance is

$${\hat{\sigma }}^{2}=\frac{1}{n-p}{\left(y-X\hat{\beta }\right)}^{\text{T}}W\left(y-X\hat{\beta }\right).$$ (A7)

The variance-covariance matrix of the estimated regression coefficients are calculated using

$$\mathrm{var}\left(\hat{\beta }\right)={\left(X^{T}WX\right)}^{-1}{\hat{\sigma }}^{2}$$ (A8)

which is a p×p matrix with diagonal elements being the variances and off-diagonal elements being the covariance.

The fitted values for all individuals in the population are

$$\hat{y}=X\hat{\beta }=X{\left(X^{\text{T}}WX\right)}^{-1}X^{\text{T}}Wy.$$ (A9)

Let us define a hat matrix by

$$H=X{\left(X^{\text{T}}WX\right)}^{-1}X^{\text{T}}W.$$ (A10)

Therefore, the fitted values are a hat function of the observed values,

$$\hat{y}=Hy.$$ (A11)

Let us partition the sample into *K* parts (folds) and denote the number of individuals in the *k*th fold by $n_{k}.$ Define $y_{k}$ as an $n_{k}\times 1$ vector, which is a subset of *y* that contains all observations in the *k*th fold. Define $X_{k}^{}$ as the $n_{k}$ rows of matrix X corresponding to the observations in the *k*th fold. The predicted residual errors are

$$e_{k}=y_{k}-X_{k}{\hat{\beta }}_{\left(-k\right)}$$ (A12)

where

$${\hat{\beta }}_{\left(-k\right)}={\left(X_{\left(-k\right)}^{\text{T}}W_{\left(-k\right)}X_{\left(-k\right)}\right)}^{-1}X_{\left(-k\right)}^{\text{T}}W_{\left(-k\right)}y_{\left(-k\right)}$$ (A13)

are the estimated regression coefficients from the data with the $n_{k}$ observations in the *k*th fold being excluded. Let us make the following matrix decomposition,

$$X^{\text{T}}WX=X_{\left(-k\right)}^{\text{T}}W_{\left(-k\right)}X_{\left(-k\right)}+X_{k}^{\text{T}}W_{k}X_{k}.$$ (A14)

Therefore,

$$X_{\left(-k\right)}^{\text{T}}W_{\left(-k\right)}X_{\left(-k\right)}=X^{\text{T}}WX-X_{k}^{\text{T}}W_{k}X_{k}.$$ (A15)

Similarly, we can rewrite

$$X_{\left(-k\right)}^{\text{T}}W_{\left(-k\right)}y_{\left(-k\right)}=X^{T}Wy-X_{k}^{\text{T}}W_{k}y_{k}.$$ (A16)

Using Woodbury matrix identity (Woodbury 1950), we have

$$\begin{array}{l} {\left(X_{\left(-k\right)}^{\text{T}}W_{\left(-k\right)}X_{\left(-k\right)}\right)}^{-1}\\ ={\left(X^{\text{T}}WX-X_{k}^{\text{T}}W_{k}X_{k}\right)}^{-1}\\ ={\left(X^{\text{T}}WX\right)}^{-1}-{\left(X^{\text{T}}WX\right)}^{-1}X_{k}^{\text{T}}{\left[X_{k}{\left(X^{\text{T}}WX\right)}^{-1}X_{k}^{\text{T}}-W_{k}^{-1}\right]}^{-1}X_{k}{\left(X^{\text{T}}WX\right)}^{-1}\\ ={\left(X^{\text{T}}WX\right)}^{-1}+{\left(X^{\text{T}}WX\right)}^{-1}X_{k}^{\text{T}}W_{k}{\left[I-X_{k}{\left(X^{\text{T}}WX\right)}^{-1}X_{k}^{\text{T}}W_{k}\right]}^{-1}X_{k}{\left(X^{\text{T}}WX\right)}^{-1}\\ ={\left(X^{\text{T}}WX\right)}^{-1}+{\left(X^{\text{T}}WX\right)}^{-1}X_{k}^{\text{T}}W_{k}{\left(I-H_{kk}\right)}^{-1}X_{k}{\left(X^{\text{T}}WX\right)}^{-1} \end{array}$$ (A17)

where

$$H_{kk}=X_{k}{\left(X^{\text{T}}WX\right)}^{-1}X_{k}^{\text{T}}W_{k}$$ (A18)

is an $n_{k}\times n_{k}$ matrix of leverage values for the *k*th fold. This matrix is the $n_{k}\times n_{k}$ diagonal block of the hat matrix *H*. Further derivation leads to

$$\begin{array}{l} X_{k}{\left(X_{\left(-k\right)}^{\text{T}}W_{\left(-k\right)}X_{\left(-k\right)}\right)}^{-1}X^{T}Wy\\ =X_{k}{\left(X^{\text{T}}WX\right)}^{-1}X^{T}Wy+X_{k}{\left(X^{\text{T}}WX\right)}^{-1}X_{k}^{\text{T}}W_{k}{\left(I-H_{kk}\right)}^{-1}X_{k}{\left(X^{\text{T}}WX\right)}^{-1}X^{T}Wy\\ =X_{k}\hat{\beta }+H_{kk}{\left(I-H_{kk}\right)}^{-1}X_{k}\hat{\beta } \end{array}$$ (A19)

and

$$\begin{array}{l} X_{k}{\left(X_{\left(-k\right)}^{\text{T}}W_{\left(-k\right)}X_{\left(-k\right)}\right)}^{-1}X_{k}^{\text{T}}W_{k}y_{k}\\ =X_{k}{\left(X^{\text{T}}WX\right)}^{-1}X_{k}^{\text{T}}W_{k}y_{k}+X_{k}{\left(X^{\text{T}}WX\right)}^{-1}X_{k}^{\text{T}}W_{k}{\left(I-H_{kk}\right)}^{-1}X_{k}{\left(X^{\text{T}}WX\right)}^{-1}X_{k}^{\text{T}}W_{k}y_{k}\\ =H_{kk}y_{k}+H_{kk}{\left(I-H_{kk}\right)}^{-1}H_{kk}y_{k} \end{array}$$ (A20)

Therefore, the predicted residual errors are

$$\begin{array}{c} e_{k}=y_{k}-X_{k}{\hat{\beta }}_{\left(-k\right)}\\ =y_{k}-X_{k}\hat{\beta }-H_{kk}{\left(I-H_{kk}\right)}^{-1}X_{k}\hat{\beta }+H_{kk}y_{k}+H_{kk}{}_{k}{\left(I-H_{kk}\right)}^{-1}H_{kk}y_{k}\\ =y_{k}+H_{kk}y_{k}+H_{kk}{\left(I-H_{kk}\right)}^{-1}H_{kk}y_{k}-X_{k}\hat{\beta }-H_{kk}{\left(I-H_{kk}\right)}^{-1}X_{k}\hat{\beta }\\ =\left[I+H_{kk}+H_{kk}{\left(I-H_{kk}\right)}^{-1}H_{kk}\right]y_{k}-\left[I+H_{kk}{\left(I-H_{kk}\right)}^{-1}\right]X_{k}\hat{\beta } \end{array}$$ (A21)

Note that

$${\left(I-H_{kk}\right)}^{-1}=-H_{kk}^{-1}-H_{kk}^{-1}{\left(I-H_{kk}^{-1}\right)}^{-1}H_{kk}^{-1}.$$ (A22)

Therefore,

$$\begin{array}{l} H_{kk}{\left(I-H_{kk}\right)}^{-1}H_{kk}=H_{kk}\left(-H_{kk}^{-1}-H_{kk}^{-1}{\left(I-H_{kk}^{-1}\right)}^{-1}H_{kk}^{-1}\right)H_{kk}\\ =-H_{kk}-{\left(I-H_{kk}^{-1}\right)}^{-1} \end{array}$$ (A23)

The coefficient of $y_{k}$ in Equation A21 is

$$I+H_{kk}+H_{kk}{\left(I-H_{kk}\right)}^{-1}H_{kk}=I+H_{kk}-H_{kk}-{\left(I-H_{kk}^{-1}\right)}^{-1}=I+H_{kk}{\left(I-H_{kk}\right)}^{-1}$$ (A24)

which is identical to the coefficient of $X_{k}\hat{\beta }$ in Equation A21. Therefore,

$$\begin{array}{c} e_{k}=y_{k}-X_{k}{\hat{\beta }}_{\left(-k\right)}\\ =\left[I+H_{kk}+H_{kk}{\left(I-H_{kk}\right)}^{-1}H_{kk}\right]y_{k}-\left[I+H_{kk}{\left(I-H_{kk}\right)}^{-1}\right]X_{k}\hat{\beta }\\ =\left[I+H_{kk}{\left(I-H_{kk}\right)}^{-1}\right]y_{k}-\left[I+H_{kk}{\left(I-H_{kk}\right)}^{-1}\right]X_{k}\hat{\beta }\\ =\left[I+H_{kk}{\left(I-H_{kk}\right)}^{-1}\right]\left(y_{k}-X_{k}\hat{\beta }\right)\\ =\left[I+H_{kk}{\left(I-H_{kk}\right)}^{-1}\right]{\hat{e}}_{k} \end{array}$$ (A25)

We can see that the predicted residual errors are a linear function of the estimated residual errors. The next step is to simplify the linear function,

$$\begin{array}{c} I+H_{kk}{\left(I-H_{kk}\right)}^{-1}={\left(I-H_{kk}\right)}^{-1}\left(I-H_{kk}\right)+H_{kk}{\left(I-H_{kk}\right)}^{-1}\\ ={\left(I-H_{kk}\right)}^{-1}\left(I-H_{kk}+H_{kk}\right)\\ ={\left(I-H_{kk}\right)}^{-1} \end{array}$$ (A26)

Therefore, the predicted residual errors have been expressed as a simple linear function of the estimated residual errors,

$$e_{k}={\left(I-H_{kk}\right)}^{-1}{\hat{e}}_{k}.$$ (A27)

The predicted residual sum of squares (PRESS) is

$$\text{PRESS}=\sum_{k=1}^{K}e_{k}^{T}W_{k}e_{k}=\sum_{k=1}^{K}{\hat{e}}_{k}^{T}{\left(I-H_{kk}\right)}^{-1}W_{k}{\left(I-H_{kk}\right)}^{-1}{\hat{e}}_{k}.$$ (A28)

Let us define

$$\Theta_{kk}={\left(I-H_{kk}\right)}^{-1}W_{k}{\left(I-H_{kk}\right)}^{-1}.$$ (A29)

The PRESS is written as

$$\text{PRESS}=\sum_{k=1}^{K}{\hat{e}}_{k}^{T}\Theta_{kk}{\hat{e}}_{k}.$$ (A30)

The PRESS is often translated into *R*-square to represent the predictability of a model,

$${\text{R}}^{2}=1-\frac{\text{PRESS}}{\text{SST}}$$ (A31)

where SST is the total sum of squares of the response variable.

## Appendix B

### Estimated random effects

Let us define

r=y−Xβ (B1)

as the phenotypic values of the trait adjusted by the fixed effects, assuming that β is known. The estimated random effects are more appropriately called the fitted random effects. Let us define the estimated vector of random effects by

$$\tilde{r}=K{\left(K+\lambda I\right)}^{-1}r=Hr$$ (B2)

where $H=K{\left(K+\lambda I\right)}^{-1}$ is the HAT matrix, $\lambda =\sigma^{2}/\sigma_{\xi }^{2}$ is the variance ratio and K is the kinship matrix. In the main text, we used *A* in place of *K*. Here we used *K* again to be consistent with the genomic selection literature. Let us define ${\hat{e}}_{j}=r_{j}-{\tilde{r}}_{j}$ as the estimated residual error for the *j*th observation or *j*th block of observations. The predicted residual error for the *j*th block of individuals is

$$e_{j}={\left(I-H_{jj}\right)}^{-1}{\hat{e}}_{j}$$ (B3)

The purpose of this appendix is to prove Equation B3 that the predicted residual error can be obtained from the estimated residual error via the leverage value (diagonal element or diagonal block) of the HAT matrix.

Let us partition the *K* matrix into

$$K=\left[\begin{array}{cc} K_{jj} & K_{j\left(-j\right)}\\ K_{\left(-j\right)j} & K_{\left(-j\right)\left(-j\right)} \end{array}\right]$$ (B4)

where $K_{jj}$ is the *j*th diagonal element of the *K* matrix, $K_{j(-j)}$ is the *j*th row of matrix *K* that excludes the *j*th column, and $K_{\left(-j\right)\left(-j\right)}$ is the *K* matrix excluding the *j*th row and the *j*th column. Corresponding to this partitioning, matrix K+λI can also be partitioned into

$$K+\lambda I=\left[\begin{array}{cc} K_{jj}+\lambda I & K_{j\left(-j\right)}\\ K_{\left(-j\right)j} & K_{\left(-j\right)\left(-j\right)}+\lambda I \end{array}\right].$$ (B5)

The inverse of the above partitioned matrix is denoted by

$${\left(K+\lambda I\right)}^{-1}={\left[\begin{array}{cc} K_{jj}+\lambda I & K_{j\left(-j\right)}\\ K_{\left(-j\right)j} & K_{\left(-j\right)\left(-j\right)}+\lambda I \end{array}\right]}^{-1}=\left[\begin{array}{cc} C_{jj} & C_{j\left(-j\right)}\\ C_{\left(-j\right)j} & C_{\left(-j\right)\left(-j\right)} \end{array}\right]$$ (B6)

where

$$\begin{array}{l} C_{jj}={\left[\left(K_{jj}+\lambda I\right)-K_{j\left(-j\right)}{\left(K_{\left(-j\right)\left(-j\right)}+\lambda I\right)}^{-1}K_{\left(-j\right)j}\right]}^{-1}\\ C_{j\left(-j\right)}=-C_{jj}K_{j\left(-j\right)}{\left(K_{\left(-j\right)\left(-j\right)}+\lambda I\right)}^{-1}\\ C_{\left(-j\right)j}=-{\left(K_{\left(-j\right)\left(-j\right)}+\lambda I\right)}^{-1}K_{\left(-j\right)j}C_{jj}\\ C_{\left(-j\right)\left(-j\right)}={\left(K_{\left(-j\right)\left(-j\right)}+\lambda I\right)}^{-1}+{\left(K_{\left(-j\right)\left(-j\right)}+\lambda I\right)}^{-1}K_{\left(-j\right)j}C_{jj}K_{j\left(-j\right)}{\left(K_{\left(-j\right)\left(-j\right)}+\lambda I\right)}^{-1} \end{array}$$ (B7)

The estimated (fitted) value of the *j*th individual is

$${\tilde{r}}_{j}=\left[\begin{array}{cc} K_{jj} & K_{j\left(-j\right)} \end{array}\right]\left[\begin{array}{cc} C_{jj} & C_{j\left(-j\right)}\\ C_{\left(-j\right)j} & C_{\left(-j\right)\left(-j\right)} \end{array}\right]\left[\begin{array}{c} r_{j}\\ r_{-j} \end{array}\right]$$ (B8)

which is eventually expressed as

$${\tilde{r}}_{j}=K_{jj}C_{jj}r_{j}+K_{j\left(-j\right)}C_{\left(-j\right)j}r_{j}+K_{jj}C_{j\left(-j\right)}r_{-j}+K_{j\left(-j\right)}C_{\left(-j\right)\left(-j\right)}r_{-j}$$ (B9)

### Predicted random effects

The predicted value for the *j*th individual is obtained by excluding the contribution from the same individual, as expressed below,

$${\hat{r}}_{j}=K_{j\left(-j\right)}{\left[K_{\left(-j\right)\left(-j\right)}+\lambda I\right]}^{-1}r_{-j}.$$ (B10)

Let us examine the four terms in the fitted value given in Equation B9,

$$\begin{array}{l} K_{jj}C_{jj}r_{j}=K_{jj}C_{jj}r_{j}\\ K_{j\left(-j\right)}C_{\left(-j\right)j}r_{j}=-K_{j\left(-j\right)}{\left(K_{\left(-j\right)\left(-j\right)}+\lambda I\right)}^{-1}K_{\left(-j\right)j}C_{jj}r_{j}\\ K_{jj}C_{j\left(-j\right)}r_{-j}=-K_{jj}C_{jj}{\hat{r}}_{j}\\ K_{j\left(-j\right)}C_{\left(-j\right)\left(-j\right)}r_{-j}={\hat{r}}_{j}+K_{j\left(-j\right)}{\left(K_{\left(-j\right)\left(-j\right)}+\lambda I\right)}^{-1}K_{\left(-j\right)j}C_{jj}{\hat{r}}_{j} \end{array}$$ (B11)

Substituting these four terms into Equation B9, we get

$$\begin{array}{l} {\tilde{r}}_{j}=K_{jj}C_{jj}r_{j}-K_{j\left(-j\right)}{\left(K_{\left(-j\right)\left(-j\right)}+\lambda I\right)}^{-1}K_{\left(-j\right)j}C_{jj}r_{j}\\ -K_{jj}C_{jj}{\hat{r}}_{j}+{\hat{r}}_{j}+K_{j\left(-j\right)}{\left(K_{\left(-j\right)\left(-j\right)}+\lambda I\right)}^{-1}K_{\left(-j\right)j}C_{jj}{\hat{r}}_{j}. \end{array}$$ (B12)

Note that the fitted random effect for the *j*th individual has been expressed as a linear function of the predicted random effect.

### Estimated and predicted errors

Let us define ${\hat{e}}_{j}=r_{j}-{\tilde{r}}_{j}$ as the estimated error and $e_{j}=r_{j}-{\hat{r}}_{j}$ as the predicted error. We then define

$$\begin{array}{l} r_{j}-{\tilde{r}}_{j}=r_{j}-K_{jj}C_{jj}r_{j}+K_{j\left(-j\right)}{\left(K_{\left(-j\right)\left(-j\right)}+\lambda I\right)}^{-1}K_{\left(-j\right)j}C_{jj}r_{j}\\ +K_{jj}C_{jj}{\hat{r}}_{j}-{\hat{r}}_{j}-K_{j\left(-j\right)}{\left(K_{\left(-j\right)\left(-j\right)}+\lambda I\right)}^{-1}K_{\left(-j\right)j}C_{jj}{\hat{r}}_{j}. \end{array}$$ (B13)

After a few steps of manipulation, we have

$$r_{j}-{\tilde{r}}_{j}=\left[I-K_{jj}C_{jj}+K_{j\left(-j\right)}{\left(K_{\left(-j\right)\left(-j\right)}+\lambda I\right)}^{-1}K_{\left(-j\right)j}C_{jj}\right]\left(r_{j}-{\hat{r}}_{j}\right).$$ (B14)

Therefore, the estimated and predicted errors have the following relationship,

$${\hat{e}}_{j}=\left[I-\left(K_{jj}C_{jj}-K_{j\left(-j\right)}{\left(K_{\left(-j\right)\left(-j\right)}+\lambda I\right)}^{-1}K_{\left(-j\right)j}C_{jj}\right)\right]e_{j}.$$ (B15)

We want to prove that the *j*th diagonal element of the HAT matrix (the leverage value for observation *j*) is

$$H_{jj}=K_{jj}C_{jj}-K_{j\left(-j\right)}{\left(K_{\left(-j\right)\left(-j\right)}+\lambda I\right)}^{-1}K_{\left(-j\right)j}C_{jj}$$ (B16)

which leads to

$${\hat{e}}_{j}=\left(I-H_{jj}\right)e_{j}.$$ (B17)

As a result,

$$e_{j}={\left(I-H_{jj}\right)}^{-1}{\hat{e}}_{j}.$$ (B18)

We now go back to the HAT matrix to see what $H_{jj}$ is. Using partitioned matrix, we have

$$\begin{array}{c} H=\left[\begin{array}{cc} K_{jj} & K_{j\left(-j\right)}\\ K_{\left(-j\right)j} & K_{\left(-j\right)\left(-j\right)} \end{array}\right]\left[\begin{array}{cc} C_{jj} & C_{j\left(-j\right)}\\ C_{\left(-j\right)j} & C_{\left(-j\right)\left(-j\right)} \end{array}\right]\\ =\left[\begin{array}{cc} K_{jj}C_{jj}+K_{j\left(-j\right)}C_{\left(-j\right)j} & K_{jj}C_{jj}C_{j\left(-j\right)}+K_{j\left(-j\right)}C_{\left(-j\right)\left(-j\right)}\\ K_{\left(-j\right)j}C_{jj}+K_{\left(-j\right)\left(-j\right)}C_{\left(-j\right)j} & K_{\left(-j\right)j}C_{j\left(-j\right)}+K_{\left(-j\right)\left(-j\right)}C_{\left(-j\right)\left(-j\right)} \end{array}\right] \end{array}$$ (B19)

Therefore,

$$H_{jj}=K_{jj}C_{jj}+K_{j\left(-j\right)}C_{\left(-j\right)j}.$$ (B20)

From Equation B11, we know

$$K_{j\left(-j\right)}C_{\left(-j\right)j}=-K_{j\left(-j\right)}{\left(K_{\left(-j\right)\left(-j\right)}+\lambda I\right)}^{-1}K_{\left(-j\right)j}C_{jj}.$$ (B21)

Substituting Equation B21 into Equation B20 yields

$$K_{jj}C_{jj}-K_{j\left(-j\right)}{\left(K_{\left(-j\right)\left(-j\right)}+\lambda I\right)}^{-1}K_{\left(-j\right)j}C_{jj}$$ (B22)

which is exactly the same as Equation B16; thus, we conclude the derivation of Equation B18. Unlike the HAT matrix in fixed models, the random model HAT matrix is not idempotent, although it remains symmetric.

The PRESS is now defined as

$$\text{PRESS}=\sum_{j=1}^{n}e_{j}^{T}e_{j}=\sum_{j=1}^{n}{\hat{e}}_{j}^{T}{\left(I-H_{jj}\right)}^{-2}{\hat{e}}_{j}.$$ (B23)

If the residual errors are heterogeneous with $e∼N\left(0,R\sigma^{2}\right)$ where R is a known diagonal matrix, all the above derivations apply except that we have to replace λI by λR in all occurrences. In addition, the PRESS should be modified as a weighted PRESS,

$$\text{PRESS}=\sum_{j=1}^{n}e_{j}^{T}W_{j}^{}e_{j}=\sum_{j=1}^{n}{\hat{e}}_{j}^{T}{\left(I-H_{jj}\right)}^{-1}W_{j}{\left(I-H_{jj}\right)}^{-1}{\hat{e}}_{j}$$ (B24)

where $W_{j}=R_{j}^{-1}$ is the weight for the *j*th observation or the *j*th block of observations.
