# Supplementary material for: Predicted Residual Error Sum of Squares of Mixed Models: An Application for Genomic Prediction
Source: G3 (Bethesda). 2017 Jan 19;7(3):895–909. doi: 10.1534/g3.116.038059 (PMC5345720; doi:10.1534/g3.116.038059)
Supplement: Supplementary file 2 [file 895FileS1.docx]

**File S1:** “mixedFunction.R” This code defines a function called mixed(). This function is used to estimate parameters of the mixed model using either the ML or the REML method along with the eigen-decomposition algorithm. The parameters include fixed effects (beta), polygenic variance (va) and residual variance (ve). In all the R codes, the variance ratio is defined as $\lambda_{\text{CODE}}=\sigma_{\xi}^{2}/\sigma^{2}$, which is the inverse of the one described in the text $\lambda_{\text{TEXT}}=\sigma^{2}/\sigma_{\xi}^{2}$. (.zip, 1 KB)

Available for download as a .zip file at:

http://www.g3journal.org/lookup/suppl/doi:10.1534/g3.116.038059/-/DC1/FileS1.zip
