# Supplementary material for: Predicted Residual Error Sum of Squares of Mixed Models: An Application for Genomic Prediction
Source: G3 (Bethesda). 2017 Jan 19;7(3):895–909. doi: 10.1534/g3.116.038059 (PMC5345720; doi:10.1534/g3.116.038059)
Supplement: Supplementary file 4 [file 895FileS2.docx]

File S2: “mixedBlupFunction.R” This code defines a function called mixedBlup(). This function is used to estimate parameters of the mixed model and perform BLUP prediction. If heritability is provided, the mixedBlup() function will only perform prediction. (.zip, 2 KB)

Available for download as a .zip file at:

http://www.g3journal.org/lookup/suppl/doi:10.1534/g3.116.038059/-/DC1/FileS2.zip
