# Supplementary material for: Predicted Residual Error Sum of Squares of Mixed Models: An Application for Genomic Prediction
Source: G3 (Bethesda). 2017 Jan 19;7(3):895–909. doi: 10.1534/g3.116.038059 (PMC5345720; doi:10.1534/g3.116.038059)
Supplement: Supplementary file 8 [file 895FileS4.docx]

**File S4:** “mixedHAT.R” This is an R program to perform mixed model analysis and HAT prediction for the inbred rice population. It takes “RIL-phe.csv” and “RIL-kk.csv” as the input data and generates three data.frames as the outputs. You need to source the R function described in R code 1. The first data.frame (fit) is the result of the mixed model analysis, including estimated fixed effects (beta), polygenic variance (va) and residual variance (ve). The second data.frame (PRED) stores the predicted phenotypic values and the predicted random effects (polygenic effects) from the HAT method along with the original observed phenotypes and other information. The third data.frame (PRESS) stores the PRESS value and the R2 (predictability) resulted from the HAT prediction. Again, you should use the sample data to test the program before analyzing your own. Be sure to define the paths of the input and output data correctly! (.zip, 1 KB)

Available for download as a .zip file at:

http://www.g3journal.org/lookup/suppl/doi:10.1534/g3.116.038059/-/DC1/FileS4.zip
