# Supplementary material for: Predicted Residual Error Sum of Squares of Mixed Models: An Application for Genomic Prediction
Source: G3 (Bethesda). 2017 Jan 19;7(3):895–909. doi: 10.1534/g3.116.038059 (PMC5345720; doi:10.1534/g3.116.038059)
Supplement: Supplementary file 10 [file 895FileS5.docx]

**File S5:** “RIL-phe.csv” has 210 rows (excluding the heater) and nine columns. The first column is the ID of the lines. The second column is the fold ID used for cross validation analysis. Columns 3 – 9 are the phenotypic values of seven traits of the inbred rice. (.csv, 12 KB)

Available for download as a .csv file at:

http://www.g3journal.org/lookup/suppl/doi:10.1534/g3.116.038059/-/DC1/FileS5.csv
