# Supplementary material for: Predicted Residual Error Sum of Squares of Mixed Models: An Application for Genomic Prediction
Source: G3 (Bethesda). 2017 Jan 19;7(3):895–909. doi: 10.1534/g3.116.038059 (PMC5345720; doi:10.1534/g3.116.038059)
Supplement: Supplementary file 12 [file 895FileS6.docx]

File S6: “RIL-kk.csv” is the kinship matrix calculated from the whole genome marker data. This file has 210 rows (excluding the header) and 212 columns. The first and second columns are required by the MIXED procedure of SAS but are not needed by the R programs. In the R codes that require the kinship matrix, the first two columns should be removed prior to the analysis. The kinship matrix should be an 210×210 symmetric matrix. (.csv, 515 KB)

Available for download as a .csv file at:

http://www.g3journal.org/lookup/suppl/doi:10.1534/g3.116.038059/-/DC1/FileS6.csv
